# Postexercise Hypotension Is Delayed in Men With Obesity and Hypertension

**DOI:** 10.3389/fphys.2022.819616

**Published:** 2022-03-08

**Authors:** Catherine L. Jarrett, Wesley J. Tucker, Siddhartha S. Angadi, Glenn A. Gaesser

**Affiliations:** ^1^Geriatric Research, Education, and Clinical Center, Veterans Affairs Medical Center (VAMC), Salt Lake City, UT, United States; ^2^Utah Vascular Research Laboratory, University of Utah School of Medicine, Salt Lake City, UT, United States; ^3^Department of Nutrition and Food Sciences, Texas Woman’s University, Houston, TX, United States; ^4^Department of Kinesiology, School of Education and Human Development, University of Virginia, Charlottesville, VA, United States; ^5^College of Health Solutions, Arizona State University, Phoenix, AZ, United States

**Keywords:** blood pressure, exercise, body mass index, obese, overweight

## Abstract

**Background:**

Postexercise hypotension (PEH) can play a major role in the daily blood pressure management among individuals with hypertension. However, there are limited data on PEH in persons with obesity and hypertension, and no PEH data in this population beyond 90 min postexercise.

**Purpose:**

The purpose of this study was to determine if PEH could be elicited in men with obesity and hypertension during a 4-h postexercise measurement period.

**Methods:**

Seven men [age = 28 ± 4 years; body mass index = 34.6 ± 4.8 kg/m^2^; brachial systolic blood pressure (SBP): 138 ± 4 mmHg; brachial diastolic BP (DBP): 80 ± 5 mmHg; central SBP: 125 ± 4 mmHg; central DBP: 81 ± 8 mmHg] performed two exercise sessions on a cycle ergometer, each on a separate day, for 45 min at ∼65% VO_2max_. One exercise session was performed at a cadence of 45 RPM and one at 90 RPM. Blood pressure was monitored with a SunTech Oscar2 ambulatory blood pressure monitor for 4 h after both exercise sessions, and during a time-matched control condition.

**Results:**

Both brachial and central SBP were not changed during the first h postexercise but were reduced by ∼5–11 mmHg between 2 and 4 h postexercise (*p* < 0.05) after both exercise sessions. Brachial and central DBP were elevated by ∼5 mmHg at 1 h postexercise (*p* < 0.05) but were ∼2–3 mmHg lower compared to control at 4 h postexercise, and ∼2–4 mmHg lower at 3 h postexercise compared to baseline. Mean arterial pressure (MAP) was elevated compared to control at 1 h postexercise after both exercise sessions, but was ∼2–3 mmHg lower compared to control at 2, 3, and 4 h postexercise, and ∼4–7 mmHg lower at 3 h postexercise compared to baseline.

**Conclusion:**

Despite the small sample size and preliminary nature of our results, we conclude that PEH is delayed in men with obesity and hypertension, but the magnitude and duration of PEH up to 4 h postexercise is similar to that reported in the literature for men without obesity and hypertension. The PEH is most pronounced for brachial and central SBP and MAP. The virtually identical pattern of PEH after both exercise trials indicates that the delayed PEH is a reproducible finding in men with obesity and hypertension.

## Introduction

Hypertension is a leading risk factor for cardiovascular disease (Whelton et al., 2018), and physical activity can lower blood pressure and reduce the rate of conversion from elevated blood pressure (BP) to hypertension (Faselis et al., 2012; Diaz and Shimbo, 2013; Pescatello et al., 2015). A single bout of exercise has been shown to lower blood pressure for at least 12 h (Pescatello and Kulikowich, 2001; Pescatello et al., 2004), and this postexercise hypotension (PEH) is thought to play an important role in the antihypertensive effects of exercise (Hamer, 2006; Brito et al., 2018).

Although PEH has been well-established in normal weight and overweight individuals, whether obesity influences the PEH response is unclear. This is mainly due to a limited number of publications on PEH in persons with obesity (Figueroa et al., 2007; Zeigler et al., 2018; Bunsawat et al., 2021). One meta-analyses reported that PEH was inversely related to body mass index [BMI (Body Mass Index); kg/m^2^], with a regression line that predicted no PEH above a BMI of approximately 31 (Carpio-Rivera et al., 2016). This meta-analysis included only one study that reported postexercise BP responses in persons with obesity. In that study, which included obese women with and without type 2 diabetes, 20 min of exercise at 65% of maximal oxygen uptake (VO_2max_) reduced systolic BP (SBP) at 10 and 20 min postexercise, but PEH was no longer evident at 30 min postexercise, which was the last measurement time (Figueroa et al., 2007). It was recently reported that 1 h of exercise at 60% of VO_2max_ produced a modest PEH of ∼2–4 mmHg in adults with obesity (Bunsawat et al., 2021). In that study, the PEH observed at 30 and 60 min postexercise was no longer present at 90 min. We previously reported that PEH was not observed in 8 men with obesity after a 40-min exercise bout at 65–70% of heart rate maximum (Zeigler et al., 2018). However, we measured blood pressure for only 1 h postexercise. Despite the lack of PEH during the 1-h postexercise measurement period, there was a clear trend for declining BP between the first 30 min postexercise and the last 30 min postexercise. This was evident for SBP, diastolic BP (DBP) and mean arterial pressure (MAP). But because BP was elevated immediately postexercise, despite the trend for decreasing BP during the 60-min postexercise measurement period, PEH relative to baseline BP was not observed. Thus, a PEH may have been observed if the postexercise measurement period been extended beyond 1 h. Because PEH can be observed for several hours after exercise (Pescatello and Kulikowich, 2001; Pescatello et al., 2004; Angadi et al., 2015), it is possible that PEH is delayed in obese individuals. In the few prior studies published on PEH in adults with obesity (Figueroa et al., 2007; Zeigler et al., 2018; Bunsawat et al., 2021), postexercise BP measurements did not extend beyond 90 min.

Another limitation of the studies on PEH in obesity is that the subject populations had relatively normal blood pressures, with mean SBP of 110 ± 10 mmHg (Bunsawat et al., 2021), 122 ± 6 mmHg (Figueroa et al., 2007), and 126 ± 7 mmHg (Zeigler et al., 2018). The magnitude of PEH is positively related to the baseline BP prior to exercise (Brito et al., 2018). Indeed, PEH has been repeatedly demonstrated in hypertensive populations (Legramante et al., 2002; Melo et al., 2006; Brito et al., 2011; Dos Santos et al., 2014; Cunha et al., 2016; Cunha et al., 2017; Ferrari et al., 2017; Imazu et al., 2017; Cunha et al., 2018; Goessler et al., 2018; de Freitas Brito et al., 2019; Pires et al., 2020; Iellamo et al., 2021). However, in virtually all of these studies the hypertensive populations included individuals with and without obesity, with mean BMI < 30 kg/m^2^ in most instances. One study reported PEH in 8 hypertensive women, all with BMI > 30 kg/m^2^ (Cunha et al., 2018), but the results may have limited applicability due to the nature of the experimental design. The exercise involved 45 min of water aerobics, and it has been demonstrated that water immersion induces significant hemodynamic changes even without exercise (Sramek et al., 2000; Stocks et al., 2004). This is especially relevant because the control condition included no water immersion and the postexercise measurement period lasted only 30 min. Consequently, a research gap exists with regard to PEH in adults with both obesity and hypertension. Thus, the purpose of this study was to determine whether PEH is observed during a postexercise period lasting 4 h in adult men with obesity and hypertension. Based on results from our previous study described above (Zeigler et al., 2018), we hypothesized that PEH would be delayed, with significantly reduced brachial and central BPs observed only after the first 1 h postexercise.

## Materials and Methods

### Participants

Seven physically inactive men (age 28 ± 4 years; BMI 34.6 ± 4.8 kg/m^2^) participated in this study. Subject characteristics are provided in Table 1. Physical activity levels were ascertained with the International Physical Activity Questionnaire (Hagstromer et al., 2006). Subjects were not currently participating in any structured exercise program or adhering to any specific diet or weight loss program. Those with known cardiovascular, pulmonary, and renal or metabolic diseases, current smokers, or those on vasoactive medications for the treatment of blood pressure, were excluded. The study was approved by the Arizona State University Institutional Review Board and was conducted in a manner consistent with the Declaration of Helsinki. Written and informed consent was obtained from each subject prior to enrollment.

**TABLE 1:** Participant characteristics.

	Mean ± SD	Range
N	7	
Age (year)	28 ± 4	20–35
Height (cm)	175.1 ± 7.4	165.0–187.5
Weight (kg)	105.8 ± 15.4	91.7–137.9
BMI (kg/m^2^)	34.6 ± 4.8	28.9–43.2
Body fat (%)	37.1 ± 4.4	31.3–43.0
Visceral fat (g)	1,398 ± 707	506–2,559
Systolic blood pressure (mmHg)	138 ± 4	131–145
Diastolic blood pressure (mmHg)	80 ± 5	70–87
VO_2max_ (ml/kg/min)	28.7 ± 6.1	18.7–36.3

*BMI, Body Mass Index; VO_2max_, maximal oxygen uptake.*

The seven subjects included in this study were part of a larger study in which glucose tolerance was the primary outcome, with the goal of determining whether muscle contraction frequency (defined by different pedaling cadences, described below) during an aerobic exercise session affected postexercise glucose tolerance (unpublished data not shown). Postexercise blood pressure was a secondary outcome in this study. Although blood pressure was not one of the inclusion/exclusion criteria, all seven men had resting SBP > 130 mmHg (see Table 1), thus meeting the definition of hypertension (Whelton et al., 2018). One of the subjects had a BMI of 28.9 kg/m^2^. However, results were essentially the same with and without including this subject in the statistical analyses (see Section “Results”).

### VO_2max_ Assessment

Prior to the PEH protocol (described below) each participant performed a ramp-style maximal exercise test on a cycle ergometer (Viasprint 150P; Ergoline, Bitz, Germany) for determination of VO_2max_. Pulmonary ventilation and gas exchange were measured continuously with a Parvo Medics TrueOne 2400 (Parvo Medics, Sandy, UT, United States). Standard three-point calibration was performed before each test. Heart rate was measured with a Polar heart rate monitor (Polar, Lake Success, NY, United States). After a 5-min warm-up phase at 50 W, power was increased by 30 W/min until exhaustion. Participants were provided with verbal encouragement throughout the test. After a cool-down period of 5–10 min, in which subjects pedaled at the warm-up work rate, each subject performed a verification phase test at a constant power of 100% of the peak power attained during the ramp test (Sawyer et al., 2015). The mean of the two highest consecutive 15-s VO_2_ averages during the ramp or verification phase tests was taken as VO_2max_. All subjects achieved a maximum respiratory exchange ratio > 1.10 on the ramp test and a heart rate > 90% of age-predicted maximum on either the ramp test or the verification phase test.

### Body Composition

Participants’ heights and weights were measured on a standard scale (Seca274, Medical Measuring Systems, Chino, CA, United States). Body composition was assessed *via* Dual-energy X-ray Absorptiometry (DEXA) (Lunar iDXA, GE Healthcare, Madison, WI, United States).

### Experimental Protocol for Determination of Postexercise Hypotension

All subjects underwent three experimental conditions in a randomized cross-over design. The conditions consisted of a non-exercise control day and two exercise conditions on a cycle ergometer at different pedaling frequencies (45 and 90 RPM). As stated above, the rationale for the two pedaling frequencies pertained to the primary outcome measure of glucose tolerance. There was no *a priori* reason for expecting cycling cadence to influence PEH. However, the two exercise trials allowed for an assessment of the reproducibility of the postexercise blood pressure responses to aerobic exercise in men with obesity. Only one study has reported information on PEH reproducibility (Fecchio et al., 2017), and that study only measured PEH at 45 min after exercise cessation.

Subjects reported to the laboratory at 7:00 a.m. for each visit, 1 h after consuming a standardized breakfast meal at home. The breakfast meal consisted of a bagel, cream cheese, and chocolate milk (630 kcal; 104 g carbohydrate; 10 g fat; 30 g protein). The same meal was consumed prior to each visit. The breakfast meal was provided to subjects the day before each laboratory visit. In addition, gift cards to a local restaurant were provided to each subject for purchasing lunch and dinner on the day before each laboratory visit. Subjects were instructed to consume the same lunch and dinner meals on the day before each laboratory visit. An ambulatory blood pressure monitor was placed on the right arm of the participant. After 15 min of seated rest, resting blood pressures were recorded. Resting blood pressure data obtained for each trial were averaged over the three trials. The average resting brachial blood pressures are presented in Table 1, and reflect the average of 5–13 total recordings taken during these baseline assessment periods for each subject. After resting blood pressure was taken, the blood pressure monitor was then set to record automatically every 15 min for 4 h during the control trial and after exercise for the two exercise trials. For exercise study visits, the monitor was removed during cycling and replaced following the cool-down. All visits were separated by at least 1 week, and this procedure was completed for all study visits.

### Aerobic Exercise

Participants performed two cycle ergometer exercise sessions on separate occasions, using the same ergometer as used for determination of VO_2max_. These exercise sessions were randomized for sequence. During one exercise session subjects maintained a cadence of 45 RPM and during the other exercise session maintained a cadence of 90 RPM. Each exercise session began with a 5-min warm-up during which time power was gradually increased so that VO_2_ was ∼65% VO_2max_ by the end of the fifth min. Subjects then exercised at this intensity for 45 min. Heart rate and VO_2_ were monitored continuously throughout each exercise session, and power was adjusted to maintain VO_2_ at ∼65% VO_2max_. Each exercise session concluded with a 5-min cool-down at 25–50 W.

### Postexercise Measurement of Blood Pressure

Blood pressure was monitored by a SunTech Oscar2, Model 250 (Sun Tech Medical, Morrisville, NC, United States) ambulatory blood pressure monitor. The Oscar 2 device has embedded, automated technology (SphygmoCor Inside™, ATCOR, Naperville, IL, United States) that allows for the estimation of central aortic systolic and diastolic pressures (Pauca et al., 2001). During the 4 h of BP measurements in the control condition and after both exercise sessions, subjects spent the entire time in a private office room adjacent to the laboratory. During this time the subjects spent most of their time seated at a desk where they were allowed to use their laptop computer or phone, or to read. The only time they were allowed to leave the private office space was to use the restroom. Participants were instructed to remain still and not talk while the device was inflating. During the 4-h postexercise period, the majority of measurements were taken while participants were seated quietly. If they were walking or standing when a measurement started (e.g., walking to the restroom) they were instructed to stand quietly until the measurement was completed. Blood pressure data were downloaded using AccuWin Pro v4.0.

### Statistical Methods

Blood pressure data were pooled into hourly averages. All statistical procedures were performed using SPSS (SPSS 23, IBM Corporation, Armonk, NY, United States). Values were tested for normality and homogeneity. One-way analysis of variance was used to test for differences in baseline values between the three trials for all blood pressures. Paired-samples *t*-tests were used detect differences in work rate, VO_2_ and heart rate responses to the two RPM conditions. Linear mixed models were used to detect differences for hourly differences for BP variables with both fixed and random effects explored. Baseline BP was included as a covariate in the linear models analysis. Pairwise comparisons were also used to determine within-condition differences in BP at each postexercise time point compared with pre-exercise baseline BP. The Bonferroni adjustment was used for multiple comparisons when appropriate. The mixed model estimated marginal means (EMM) are shown in all figures. A *p*-value < 0.05 was considered statistically significant.

## Results

The mean VO_2_ (45 RPM = 1.93 ± 0.30 L/min; 90 RPM = 1.97 ± 0.33 L/min), and heart rate (45 RPM = 147 ± 12 beats/min; 90 RPM = 152 ± 17 beats/min) were not different between exercise conditions. However, the power output during exercise at 45 RPM (106 ± 27 W) was significantly greater (*p* < 0.001) than that during exercise at 90 RPM (90 ± 30 W). The lower power output during exercise at the faster cadence was necessary due to the effect of pedaling frequency on VO_2_ (Gaesser and Brooks, 1975).

**FIGURE 1:**
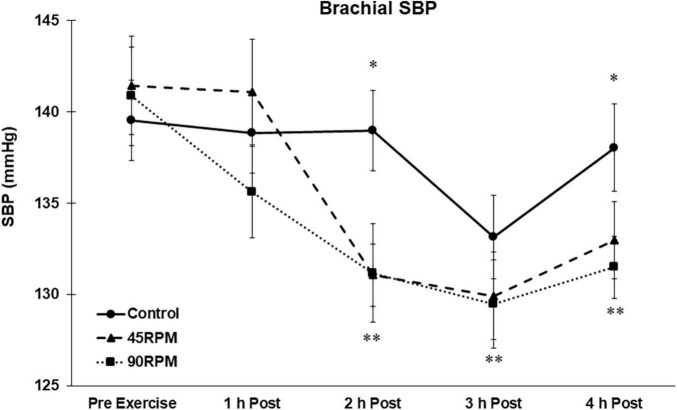
Brachial systolic blood pressure (SBP) postexercise with time matched control. Data presented as Estimated Marginal Means ± SE; *N* = 7. *Significant difference between control and exercise conditions *p* < 0.05. **Significantly different from baseline for both exercise conditions *p* < 0.05.

**FIGURE 2:**
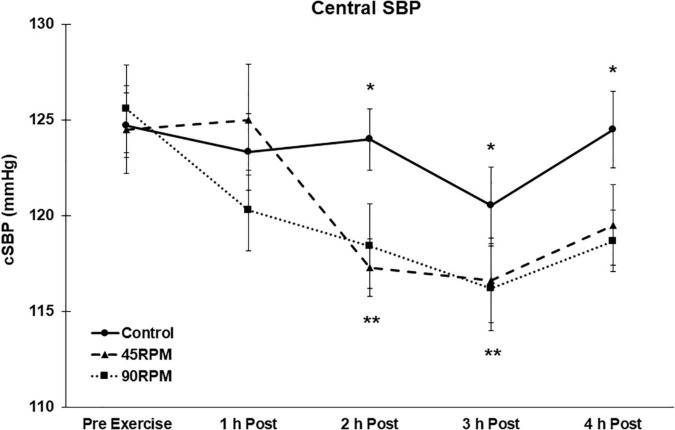
Central systolic blood pressure (cSBP) postexercise with time matched control. Data presented as Estimated Marginal Means ± SE; *N* = 7. *Significant difference between control and exercise conditions *p* < 0.05. **Significantly different from baseline for both exercise conditions *p* < 0.05.

There were no differences in baseline blood pressures for the three conditions. All blood pressures remained unchanged during the non-exercise control trial. Brachial and central SBPs were unchanged during the first h postexercise. Compared to corresponding control values, brachial SBP was ∼5–8 mmHg lower at 2 and 4 h postexercise, and central SBP was ∼5–7 mmHg lower at 2, 3, and 4 h postexercise (*p* < 0.05; Figures 1, 2). Compared to baseline, brachial SBP was reduced by ∼8–11 mmHg at 2, 3, and 4 h postexercise, and central SBP was reduced by 5–10 mmHg at 2 and 3 h postexercise (*p* < 0.05).

**FIGURE 3:**
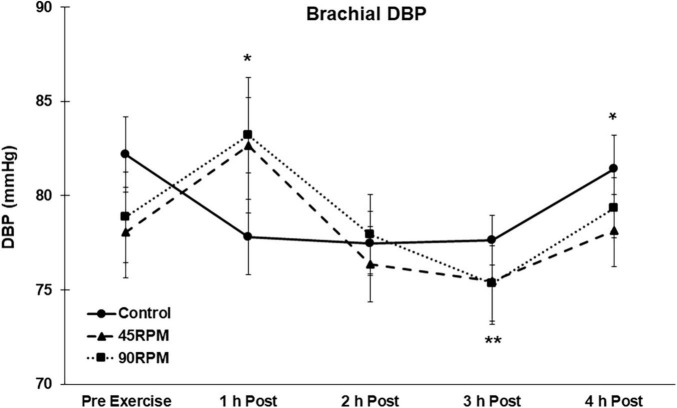
Brachial diastolic blood pressure (DBP) postexercise with time matched control. Data presented as Estimated Marginal Means ± SE; *N* = 7. *Significant difference between control and exercise conditions *p* < 0.05. **Significantly different from baseline for both exercise conditions *p* < 0.05.

**FIGURE 4:**
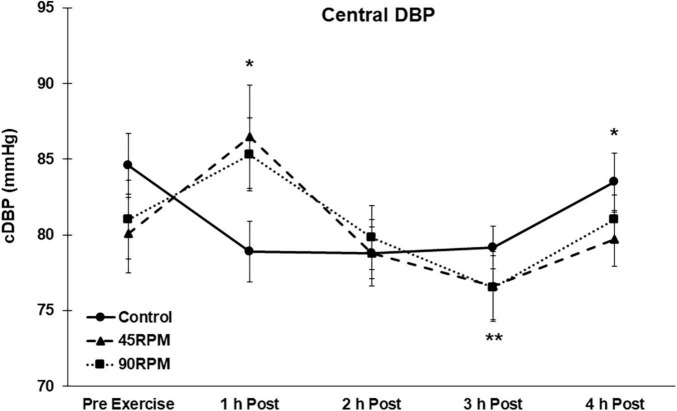
Central diastolic blood pressure (cDBP) postexercise with time matched control. Data presented as Estimated Marginal Means ± SE; *N* = 7. *Significant difference between control and exercise conditions *p* < 0.05. **Significantly different from baseline for both exercise conditions *p* < 0.05.

During the first h postexercise, brachial DBP was ∼5 mmHg higher, and central DBP ∼6–8 mmHg higher, compared to the corresponding control value (*p* < 0.05; Figures 3, 4). Thereafter, the only significant difference in DBP was a lower brachial and central DBP at 4 h postexercise compared to the control trial. Compared to baseline, both brachial and central DBP were reduced by ∼2–4 mmHg only at 3 h postexercise (*p* < 0.05).

**FIGURE 5:**
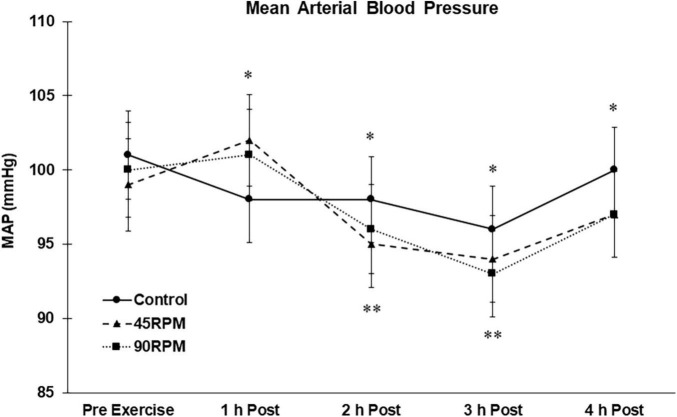
Mean arterial blood pressure (MAP) postexercise with time matched control. Data presented as Estimated Marginal Means ± SE; *N* = 7. *Significant difference between control and exercise conditions *p* < 0.05. **Significantly different from baseline for both exercise conditions *p* < 0.05.

Compared to the control trial, mean arterial pressure was elevated at 1 h postexercise after both exercise sessions but was ∼2–3 mmHg lower compared to control at 2, 3, and 4 h postexercise (*p* < 0.05; Figure 5). Compared to baseline, MAP was reduced by ∼4–7 mmHg at 2 and 3 h postexercise (*p* < 0.05).

Removal of the subject with a BMI of 28.9 kg/m^2^ did not materially affect the results. Brachial SBP was also significantly lower at 3 h postexercise for both 45 and 90 RPM, whereas it was not with *n* = 7. With *n* = 6, brachial and central DBP were significantly lower at 3 h postexercise, but no longer significant at 4 h postexercise. Results for MAP were unchanged with *n* = 6.

## Discussion

The main finding of this study was that PEH is observed in obese men, but the blood pressure-lowering effect of exercise is not observed until after the first h postexercise. Furthermore, due to the fact that the PEH response in our subjects was observed after both exercise trials that differed only in terms of cycling cadence, our results demonstrate that the delayed PEH response in men with obesity is reproducible.

Although PEH has been consistently reported in non-obese populations (Pescatello and Kulikowich, 2001; MacDonald, 2002; Forjaz et al., 2004; Jones et al., 2008; Angadi et al., 2010; Liu et al., 2012; Terblanche and Millen, 2012; Halliwill et al., 2013; Hecksteden et al., 2013; Brito et al., 2014; Angadi et al., 2015; Carpio-Rivera et al., 2016; Fecchio et al., 2017; Brito et al., 2018; Zeigler et al., 2018), there are limited data published on individuals with obesity (Figueroa et al., 2007; Zeigler et al., 2018; Bunsawat et al., 2021). Although it has been reported that PEH is not associated with BMI (Hamer and Boutcher, 2006), that study only included men identified as normal weight (BMI = 23.0 ± 1.7 kg/m^2^) or overweight (BMI = 27.5 ± 1.4 kg/m^2^). A meta-analysis reported a significant correlation between BMI and PEH (*r* = 0.26, *p* < 0.001), with a regression line predicting no PEH for individuals with BMI greater than ∼31 kg/m^2^ (Carpio-Rivera et al., 2016). Our data are not consistent with that prediction, as a significant PEH was evident for 2–4 h postexercise in our subjects. To our knowledge, this is the first time a PEH has been reported in individuals with obesity during this postexercise time period.

In an earlier study of 12 subjects with BMI 25–35 kg/m^2^ (mean = 29 ± 4 kg/m^2^), a PEH was observed 1 h after a maximal exercise test (Hecksteden et al., 2013). SBP was reduced from 134 ± 18 to 125 ± 13 mmHg, and DBP reduced from 88 ± 10 to 84 ± 7 mmHg 1 h after the maximal exercise test. Information on the number of participants with BMI > 30 kg/m^2^ was not provided. In a recent study of young adults with and without obesity, it was reported that 1 h of moderate-intensity cycling at 60% VO_2peak_ reduced brachial SBP by ∼2 mmHg and central SBP by ∼3–4 mmHg, with no differences between subjects with BMI < 25 kg/m^2^ and subjects with BMI > 30 kg/m^2^ (Bunsawat et al., 2021). In that study, PEH was significant at 30 and 60 min postexercise but was not evident by 90 min postexercise. Because this study did not include a control condition, PEH was assessed relative to pre-exercise baseline blood pressure. Even if diurnal variation in blood pressure could be expected to be minimal over the 90-min postexercise period, this could still have influenced the interpretation of PEH effect of exercise due to the small PEH observed (e.g., ∼2 mmHg PEH for brachial SBP). Including a non-exercise control trial for PEH assessment is essential for determination of the true impact of exercise on PEH due to the inherent fluctuations in resting BP over several h (see Figures 1– 4).

Body mass index has been reported to be unrelated to PEH (Hamer and Boutcher, 2006), and also inversely related to PEH (Carpio-Rivera et al., 2016). However, there were virtually no subjects with BMI > 30 in these studies. In a previous manuscript we reported that PEH was not observed in obese men (Zeigler et al., 2018). However, postexercise BP was only measured for 1 h. Furthermore, in our previous study it was evident that the pattern of BP responses during the 1 h postexercise was such that a PEH might have eventually been observed with a longer postexercise measurement period. Our current postexercise data are consistent with that finding because postexercise BP was also not observed during the first hour after exercise, but a significant PEH was observed between 2 and 4 h postexercise. The magnitude of the PEH for SBP in our subjects, ∼5–8 mmHg at 2 and 4 h postexercise compared to control and ∼5–11 mmHg at 2–4 h postexercise compared to pre-exercise baseline values, is at least as great as that observed in non-obese individuals during this same time period (Angadi et al., 2015). The transient increase in brachial and central DBP at 1 h was not expected. To our knowledge, this is the first time that a significant increase in brachial and central DBPs have been reported after aerobic exercise. The fact that DBP was significantly elevated at 1 h postexercise after both exercise trials suggests that this was not a spurious finding.

The reductions in postexercise central blood pressures may be of particular clinical significance because data from the Conduit Artery Function Evaluation study demonstrated significant and divergent effects of central vs. brachial blood pressure lowering (Williams et al., 2006). Specifically, a reduced central aortic SBP of ∼4 mmHg was associated with significantly lower total cardiovascular events and/or procedures as well as development of renal impairment. Further, it is important to note that reductions in central blood pressures are superior to reductions in brachial blood pressures with regard to predicting improved subclinical outcomes such as left ventricular hypertrophy, carotid IMT, and urinary albumin secretion (Kollias et al., 2016). We acknowledge that these reports on the significance of central BP reflect measurements under resting conditions, but PEH may contribute to the overall antihypertensive effects of exercise (Hamer, 2006; Brito et al., 2018).

Most studies of PEH have reported measurements only for the initial hour after exercise (Marcal et al., 2021). Thus it is difficult to compare our subjects’ PEH responses over longer postexercise periods with published data. We previously reported brachial blood pressures during a 3-h postexercise period after continuous and interval exercise in young, non-obese adults (Angadi et al., 2015). The greatest PEH was observed during the first h postexercise, and averaged ∼4–6 mmHg. However, the PEH was no longer evident by the third hour postexercise after the steady-state continuous bout of exercise, which was comparable to the exercise bouts in the present study. By contrast, in the current study both brachial and central SBP and DBP were significantly lower than the corresponding control values 4 h after exercise. This 4-h PEH was observed for both exercise trials (Figures 1–4). In fact, the peak PEH occurred at approximately 3 h postexercise, at a time when PEH was no longer evident after a similar continuous exercise session in subjects without obesity (Angadi et al., 2015). One difference is that the exercise duration in the current study was 45 min, compared to 30 min for our previous study. Although some studies have shown that exercise duration had no effect on PEH (MacDonald et al., 2000; Guidry et al., 2006), a more recent meta-analysis reported that exercise duration was inversely correlated (*r* = −0.19; *P* = 0.01) with the change in postexercise SBP (Carpio-Rivera et al., 2016). The fact that a pronounced PEH was still apparent 4 h postexercise in the current study suggests that PEH, although delayed, may extend for a longer period of time in adult men with both obesity and hypertension.

### Strengths and Weaknesses

One important strength of our study is the fact that the PEH was essentially identical after both exercise sessions. Our study was designed primarily to determine whether exercise of the same intensity and duration, but differing in muscle contraction rate, would differentially affect postexercise glucose tolerance. Blood pressure responses were a secondary outcome. By having two exercise sessions that were the same in intensity and duration, the second exercise session served as a *de facto* reliability test. The observation that postexercise BP responses were the same for both exercise tests indicates that our PEH findings in men with obesity and hypertension are reproducible.

One weakness is that our sample size is relatively small, and included only men. The magnitude of PEH has been reported to be greater in men compared to women (Carpio-Rivera et al., 2016). However, despite the small sample size, due to the consistent PEH for both exercise conditions, our results strongly suggest that the delayed PEH in men with obesity and hypertension is not a spurious finding.

Our subjects were not taking anti-hypertensive medications, whereas most studies of PEH in hypertensive individuals have included subjects on blood pressure medications (Melo et al., 2006; Brito et al., 2011; Cunha et al., 2016; Cunha et al., 2017; Ferrari et al., 2017; Imazu et al., 2017; Cunha et al., 2018; Goessler et al., 2018; de Freitas Brito et al., 2019; Costa et al., 2020; Pires et al., 2020). Thus, our results may not be applicable to those taking anti-hypertensive medications. Also, our subjects were physically inactive, and it has been documented that exercise training status affects PEH in older hypertensive adults on medication (Imazu et al., 2017; Iellamo et al., 2021). Whether training status affects PEH in younger adults with obesity and hypertension has not been evaluated.

One of our subjects had a BMI of <30 kg/m^2^. However, even when restricting our analyses to those subjects with BMI > 30 kg/m^2^, the results were essentially unchanged.

## Conclusion

Due to the small sample size our results must be viewed as preliminary. Nevertheless, they strongly suggest that PEH occurs in men with obesity and hypertension, but that the blood pressure-lowering effect of a single bout of aerobic exercise is delayed until after the first h postexercise. The PEH is most pronounced for brachial and central SBP and MAP. The magnitude of the PEH is at least as great as that observed in non-obese subjects, and may last for a longer duration.

## Data Availability Statement

The raw data supporting the conclusions of this article will be made available by the authors, without undue reservation.

## Ethics Statement

The studies involving human participants were reviewed and approved by Arizona State University Institutional Review Board. The patients/participants provided their written informed consent to participate in this study.

## Author Contributions

CJ performed the experiments. CJ and SA performed the statistical analyses. CJ and GG wrote the initial draft of the manuscript. All authors contributed to conception and design of the study, critically revised the manuscript, and approved the final version of the manuscript.

## Conflict of Interest

The authors declare that the research was conducted in the absence of any commercial or financial relationships that could be construed as a potential conflict of interest.

## Publisher’s Note

All claims expressed in this article are solely those of the authors and do not necessarily represent those of their affiliated organizations, or those of the publisher, the editors and the reviewers. Any product that may be evaluated in this article, or claim that may be made by its manufacturer, is not guaranteed or endorsed by the publisher.
